# Is a Pre-Existent Cone-Beam Computed Tomography Able to Detect Metal Dental Posts?

**DOI:** 10.3390/dj12070229

**Published:** 2024-07-22

**Authors:** Michael Solomonov, Avi Hadad, Joe Ben Itzhak, Alex Lvovsky, Hadas Azizi

**Affiliations:** 1 Department of Endodontics, Israel Defense Forces (IDF) Medical Corps, Tel Hashomer, Ramat Gan, Israel; 2 “Bina” Program, Faculty of Dental Medicine, Hebrew University of Jerusalem, 12271 Jerusalem, Israel

**Keywords:** cast post, cone-beam computed tomography, prefabricated post

## Abstract

(1) Background: In this study, the efficacy of cone-beam computed tomography (CBCT) in detecting dental posts was compared to periapical radiography. (2) Methods: A retrospective evaluation of 53 patients’ periapical radiographs and CBCT images was performed. The presence and type of the intra-canal dental post were initially determined on the periapical images (PA) radiographs’ examination and were then compared to the observer’s ability to detect the dental post on a CBCT image. The effect of the post’s type (metal cast or prefabricated) on its detection on CBCT images was determined. (3) Results: 10.5% of teeth that were identified as having a post on a PA radiograph were not identified as having a post on the CBCT examination (*p* < 0.05). Approximately 17.6% of teeth that were identified as not having a post on a PA radiograph were identified as having a post on the CBCT examination (*p* < 0.05). Moreover, 16.3% and 50% of teeth with a prefabricated or cast posts on PA radiographs were falsely identified on the CBCT examination, respectively (*p* < 0.05). (4) Conclusions: A CBCT image is an insufficient tool for the identification of metal prefabricated and cast posts. A PA image is the recommended radiographic tool for achieving information about the post-endodontic restoration status of teeth candidates for endodontic retreatment in patients with a former CBCT scan.

## 1. Introduction

### 1.1. Background

Trauma and decay frequently result in the substantial loss of tooth structure, often necessitating a root canal treatment, followed by the restoration of the lost coronal structure. Dental posts are commonly inserted into a root canal as part of the post-endodontic restoration process. In the case of insufficient coronal residual structure, an intracanal post serves as a retention tool for the core that replaces the lost coronal tooth structure [1]. Post-core systems have been used in dentistry for more than 250 years [2]. This procedure maintains the crown integrity, enabling dentistry to achieve both esthetic and functional rehabilitation.

Restoration of endodontically treated teeth necessitates a thorough understanding of the material science, defect analysis, force dynamics, and the mechanical engineering principles involved in preparation and design [3]. Thus, different materials, designs, and techniques for post restorations are available. Traditionally, posts can be classified based on their elastic modulus as follows [4]:

- Metallic posts (both prefabricated and cast metal)
- Ceramic posts
- Carbon fiber posts

The use of posts is strongly influenced by the country or even region where the dentist practices as well as the postgraduate education they have received [5].

In Israel, most general practitioners and specialists in prosthodontics prefer to use stainless steel prefabricated posts and cast posts. This preference is due to market preferences, economic factors, and the diversity of prosthodontic department guidelines.

The success rate of primary endodontic treatment ranges between 80% and 95% [6]. Endodontic retreatment or apical surgery may be suggested as treatment options for addressing post-treatment endodontic diseases, and the clinician should choose between the two based on the specific circumstances of each case [7]. The presence of a dental post is one of several factors influencing this decision. The type, length, diameter, and shape of the post may affect the clinical decision on whether to retreat through the existing fixed partial denture (FPD), conduct endodontic root canal retreatment after removing the FPD, or opt for a surgical approach without coronal disassembly [8,9,10].

A preoperative radiographic examination is essential to detect the existence, type, and other characteristics of a post in root-canal-filled teeth for guiding the appropriate treatment decision and planning. To date, periapical radiography has been the main radiographic method used for diagnosis and treatment planning prior to endodontic treatments [11,12]. The use of Cone-Beam Computed Tomographic (CBCT) imaging in dentistry is prevalent [13,14,15]. It substantially impacts endodontic decision-making, particularly in high difficulty cases [16].

Streak artifacts appear as linear hyperdensities that radiate from a metallic material and might cause significant interference when CBCT images are being reviewed [17].

### 1.2. Current Practices

According to the endodontic guidelines, the decision of whether to conduct a CBCT scan should be made after clinical and periapical (PA) radiographic examinations have been conducted. This recommendation aims to reduce the excessive use of CBCT in order to minimize patient radiation exposure, in accordance with the ‘as low as reasonably achievable’ (ALARA) principle [18]. However, clinicians may encounter situations where patients are referred for treatment with an existing CBCT image and without PA imaging. In these cases, the CBCT was flipped with the PA radiography as the primary radiographic diagnostic tool. Thus, it might be anticipated that, in these cases, there is a possible problem of misinterpretation due to the presence of dental implants, metal restorations, prosthodontic posts, and endodontic obturation materials that might create distinct “streak” artifacts [19,20,21,22]. Metal artifact reduction algorithms and image data processing methods have been developed to minimize potential CBCT image deterioration [23].

### 1.3. Research Gap

To our knowledge, no research has investigated the ability of CBCT to detect the presence or type of dental posts. This study aimed to evaluate the ability of CBCT to determine the presence and type of posts in vivo compared to PA radiographs.

## 2. Materials and Methods

This retrospective study was approved by the local medical research ethics committee (IDF Ethics Committee: IDF-1285). All methods were performed following the relevant guidelines and regulations.

A total of 500 patients’ CBCT images from the database of the maxillofacial department between 2015 and 2017 were screened. 101 teeth were selected. The inclusion criteria were teeth with previously documented digital PA images and CBCT images that had previously undergone a root canal treatment and fixed coronal restoration. Regarding the exclusion criteria, they pertain to teeth without a previous root canal treatment and teeth with previous root canal treatment but lacking a fixed coronal restoration. For all of these cases, the PA and CBCT images had previously been taken as part of their endodontic evaluation and/or implant treatment planning. The PA images were taken using a FireCR Dental Reader (FireCR Dental Reader, 3DISC, Herndon, Imaging, VA, USA) with a paralleling technique, a horizontal angle difference of about 10 degrees, and an exposure time of 0.25 s. The CBCT images (Alioth; Asahi Roentgen IND, Kyoto, Japan) were obtained at 360 X-ray tube head rotation. The scans were reformatted to standard manufacturer settings. The exposure parameters of each scan were as follows: constant tube voltage of 85 kV, tube current of 6 mA, and medium field of view (FOV) of 80 mm × 80 mm, a resolution of 0.155 × 0.155 × 0.154999 mm. CBCT exposures were performed with the minimum exposure necessary for adequate image quality.

Calibration: Two graduate endodontic residents (H.A. and A.H.) were calibrated to determine the presence of a dental post and its type by analyzing ten documented clinical cases of teeth that underwent root canal nonsurgical retreatment and coronal restoration. The cases had medical records that contained the post presence/absence and its type (prefabricated or cast). The cases were evaluated using the existing PA radiographs and CBCT images (sagittal, axial, and coronal slices) based on the same criteria and variants. Therefore, in the current investigation, PA images were utilized as the gold standard tool for identifying the existence of a post and its type. This was established through the comparison with medical records during the calibration process.

The images were analyzed simultaneously by the evaluators. In cases of disagreement, a third definitive evaluation was conducted by an endodontist with more than 15 years of experience (M.S.). The observation time was not restricted. Every evaluation included a summary of the tooth number, the presence of a post, and the type of post (prefabricated, cast, or one whose type could not be determined).

Examination of PA radiograph: The radiographs were evaluated on a computer screen (SyncMaster 245BW 24-inch LCD Monitor; Samsung, Seoul, Republic of Korea) using image evaluation software 1.0.9.3223 (CDR DICOM-5; Sirona, Schick Technologies, Long Island City, NY, USA). The observers were allowed to use the image enhancement features of the software.

The teeth were classified as follows:

1. Teeth with root canal filling, a core without a post
2. Teeth with root canal filling, a core with a stainless-steel prefabricated post
3. Teeth with root canal filling, cast post and core

Examination of CBCT images: The images were analyzed with the OnDemand3D software (CyberMed, Irvine, CA, USA) in a darkroom using a 21.3 inch flat-panel, color, active-matrix, thin-film-transistor medical display (MultiSync^®^ MD215MG; NEC, Munchen, Germany). The contrast and brightness of the images were adjusted using the image processing tool of the software to ensure optimal visualization.

The evaluators were blinded to the results obtained from the periapical radiographs.

The observers evaluated the images twice, with an interval of 4 weeks between each evaluation, to calculate the interobserver agreement. The results were recorded and submitted for statistical analysis. A chi-squared analysis was performed, with *p* < 0.05 considered as a significant result; Cohen’s kappa values were calculated for interobserver agreement evaluation. All statistical analyses were performed with the SPSS 20.0 software (IBM Corp, Armonk, NY, USA).

## 3. Results

The study analyzed the CBCT images of 53 patients aged 18–45 years (mean age = 31 years) from the database of 500 patients. 40% were males, and 60% were females. The sample included 37 molar teeth, 24 premolars, and 40 anterior teeth. Ten of the teeth in this study originated from two patients (five teeth each). Eight of the teeth originated from two other patients (four teeth each). Twenty-four of the teeth originated from eight patients (three teeth each). Thirty-four of the teeth originated from seventeen patients (two teeth each). The remaining twenty-four patients each contributed one tooth that met the inclusion criteria. Therefore, twenty-nine (54%) patients in this study contributed more than one tooth, and 13.8% of the teeth in this study were adjacent to each other. Table 1 displays the number of patients and teeth in this study.

Upon the primary radiographic examination, the PA images revealed 34 teeth with a root canal filling and a core without a dental post; 43 teeth with a root canal filling and a core with a stainless-steel prefabricated post; and 24 teeth with root canal filling and a cast post and core. However, CBCT examination images revealed 36 teeth with a root canal filling and a core without a dental post; 46 teeth with a root canal filling and a core with a stainless-steel prefabricated post; and 14 teeth with root canal filling and a cast post and core. In five teeth, the post was detected but its type could not be determined. Table 2 and Table 3 display the number of teeth that were detected with or without post on both of the radiograph methods.

Out of 10.5% of the teeth with a post that were detected on a PA image (4.5% as prefabricated, and 6% as cast posts), the post was not detected on a CBCT image (*p* < 0.05).

Out of 17.6% of the teeth without a detected post on a PA image, the post was detected upon examination on a CBCT image (8.8% as prefabricated post; 2.9% as cast post; and 5.9%, the post type could not be determined) (*p* < 0.05).

**Figure 1:**
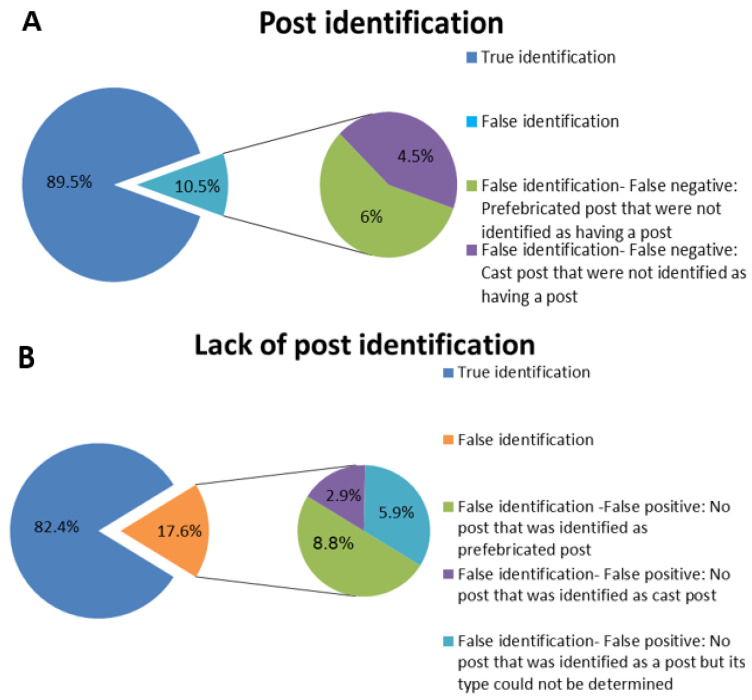
(**A**) Post identification (percentage) by CBCT imaging (*p* < 0.05); (**B**) Lack of post identification (percentage) by CBCT imaging (*p* < 0.05).

**Figure 2:**
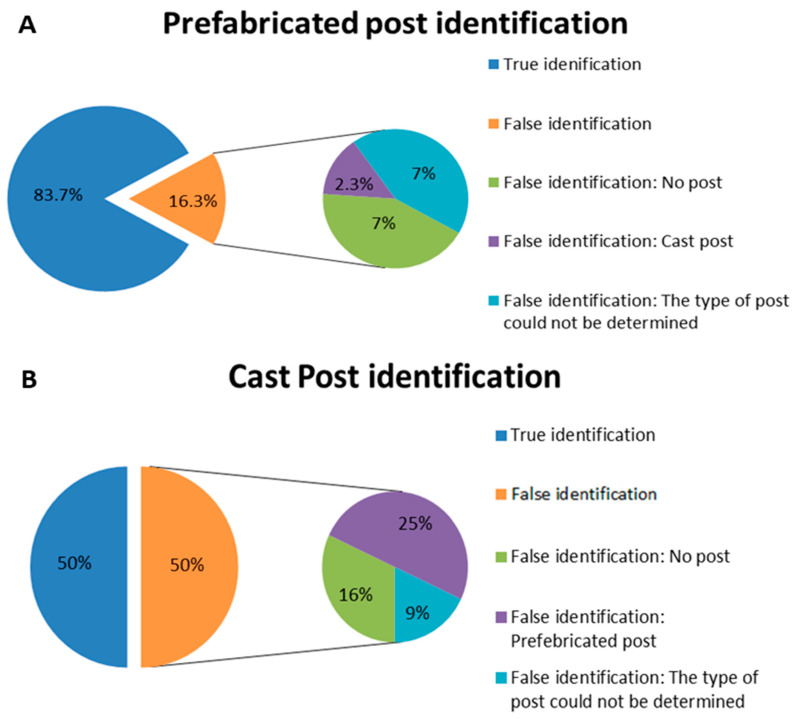
(**A**) True and false identification (percentage) of prefabricated posts by CBCT imaging (*p* < 0.05); (**B**) True and false identification (percentage) of cast posts by CBCT imaging (*p* < 0.05).

16.3% of the teeth with a detected prefabricated post on a PA image had different findings upon examination on a CBCT image (*p* < 0.05): About 7% were detected as having no post; 2.3% as a cast post; and in 7%, the post type could not be determined on a CBCT image (*p* < 0.05). Half of the teeth with a detected cast post on a PA image had different findings on a CBCT image (*p* < 0.05). 16% were detected as having no post; 25% as a prefabricated post; and in 9%, the post type could not be determined on a CBCT image (*p* < 0.05). Figure 1 and Figure 2 present the general and prefabricated and the cast posts detection on CBCT images, respectively (*p* < 0.05).

5% and 2% of the anterior and posterior teeth, respectively, with a detected post on a PA image were not detected as having a post on a CBCT image (*p* > 0.05), while 3% and 3% of the anterior and posterior teeth, respectively, without a detected post on a PA image were detected as having a post on a CBCT image (*p* > 0.05).

The Kappa value for the interobserver agreement was 0.72.

## 4. Discussion

Decision-making in post-endodontic treatment disease might be complex [24]. The choice between retreating through the existing FPD, removing the FPD for retreatment, or opting for surgical intervention depends on various factors, including the type of post-endodontic restoration [8].

### 4.1. Post Selection

Restoration of endodontically treated teeth requires a thorough understanding of material science, defect analysis, force dynamics, and mechanical engineering principles involved in preparation and design [3]. The first decision is whether to use a post at all, as its primary function is to provide retention for the core [4]. The previous belief that a post strengthens the root is no longer accepted and has been disproved. Posts can transmit occlusal forces into the root canal, reducing tooth strength, and potentially causing damage by thinning dentin, predisposing it to vertical root fractures (VRF) or causing perforations during the canal space preparation prior to their insertion. It has long been recommended not to use a post if sufficient coronal dentin is preserved after endodontic treatment. However, in many cases, a post is necessary to address insufficient coronal tissue. Additionally, in some countries, insurance companies may not cover post-endodontic restoration if no post is visible on the final X-ray.

### 4.2. Material Considerations

When a post is needed, clinicians must choose a specific type. Different materials, designs, and techniques for post restorations are available, classified based on their elastic modulus as follows: metallic posts (both prefabricated and cast metal), ceramic posts, and carbon fiber posts have high elastic modulus values, whereas glass fiber posts have low elastic modulus [4]. There is ongoing debate in the prosthodontic community regarding guidelines for post selection, specifically non-rigid metallic versus rigid non-metal posts. Although various claims have been made about nonmetallic posts, there is a need for long-term clinical evaluation of both metallic and nonmetallic post systems to allow for definitive recommendations. Fiber posts, which are more flexible than metal posts, have been commercially advocated to prevent root fractures, but this is not well supported by the literature. Excessive flexibility can undermine the core’s retention and increase the risk of secondary caries. Over the long term, fiber posts are less predictable than metal ones [25].

Another contentious matter revolves around the cementation of non-metal posts with composite cements. This is primarily due to the substantial C-factor within the canal, compounded by matrix metalloproteinase activity, which compromises bonding integrity. Additionally, the presence of residual microorganisms following chemomechanical preparation within the canal can utilize carbon from the composite as a nutritional source, deterring many practitioners from using non-metal posts [26,27,28,29,30,31].

Metallic posts continue to be the standard for most situations because they have stood the test of time [32].

Metal posts are recommended for teeth with minimal coronal dentin, as they withstand greater biting stresses. Fatigue-induced failure in this system would occur at higher stress levels and after a considerably longer time compared with a low-modulus post [32,33,34,35]. Non-rigid posts are not recommended for compromised teeth with very little tooth structure remaining above the tissue. The crown margins should engage at least 2 to 3 mm of the axial wall. The primary concern about non-metal posts is whether they allow for movement of the core during function or parafunction [36].

Custom-cast posts may be used for moderate-to-severe tooth loss and involve laboratory procedures, which demand more chair time than other posts. Titanium metal posts were introduced to address concerns about corrosion of stainless-steel posts potentially causing VRFs, though this hypothesis has not been conclusively proven. When precise dentin preservation is essential, many prosthodontists opt for thinner stainless-steel posts due to the limited availability of thin-diameter titanium posts offered by manufacturers.

There are no universally clear guidelines for selecting the appropriate post, and marketing pressures can influence a practitioner’s choice. Additionally, the country or region where a dentist practices and their postgraduate education can strongly affect the clinician’s decision to use a post and which specific post to choose [5].

In our region, most prosthodontists continue to use prefabricated stainless steel and cast posts as their primary choice in cases requiring a dental post as part of the post-endodontic restoration.

### 4.3. Post Removal

Removing a cast post through an existing FPD is generally impractical compared to removing a prefabricated post. However, the process of removing a prefabricated post may still encounter challenges affected by the cementing agent [37,38] and the post’s characteristics such as length, diameter, whether the post is parallel or tapered, and active or non-active [39,40]. Post-removal procedures may cause more cracks [41] or root fractures in rare cases [42], especially in roots with thin dentine walls.

Retreating a previous root canal treatment with a non-metal post is challenging because this type of post cannot be removed but must instead be destroyed within the root canal itself. Various types of burs and ultrasonic (US) tips have been developed for this purpose, but the risk of perforation and ledging remains prevalent in clinical practice [43,44]. Post-removal procedures primarily rely on using US tips, either with or without water cooling, depending on the type of cement used. When posts are cemented with phosphate cement, glass ionomer cement, or zinc oxide–eugenol cement, using a US device with water cooling can create micro-cracks in the cement, facilitating post removal [45]. However, in cases where composite cementation is employed, US should be used without cooling to generate heat and disrupt the composite bonding, preventing overheating of the surrounding tissues [46].

### 4.4. Radiographic Examination

Periapical radiography has been the primary method for diagnosis and treatment planning prior to endodontic treatments [11,12]. Examining radiographic imaging data, including the presence and type of a post, is essential for proper decision-making regarding suitable treatment options. Stainless-steel prefabricated serrated posts and smooth cast posts can be easily diagnosed on PA images because of their high radiopacity and clear outline, distinguishing them from the appearance of the obturated root canal. Titanium posts are radiographically similar to gutta-percha. Hence, their radiographic detection is difficult [47,48]. Most of the fiber posts are relatively radiolucent [49].

Our study did not identify any fiber or titanium posts. There is clinical significance in evaluating the ability of PA radiography and CBCT to detect both titanium and non-metal posts, particularly in countries where these types of posts are commonly used.

CBCT has become a popular tool in endodontic practice [15], allowing visualization of the dentition, maxillofacial skeleton, and surrounding anatomic structures in three dimensions [50]. CBCT significantly impacts endodontic decision-making, particularly in high-difficulty cases [16]. Several studies and case reports have demonstrated that CBCT is a better diagnostic tool than PA radiography for identifying periapical lesions [51], diagnoses and treatment planning of external cervical resorption [52], root canal anatomy, including the amounts of root canals and their configuration [53,54] and various endodontic conditions [55,56]. A high level of interobserver and intraobserver reliability in detecting apical periodontitis validates CBCT imaging as a useful diagnostic tool [57]. The effective dose of a periapical radiograph in the molar region has been reported to range from 1 to 5 µSv [58,59,60], while the effective dose of CBCT imaging ranges from 46 to 54 µSv for Scanora or Accuitomo CBCT devices [61]. The use of CBCT in endodontics is justified in specific cases when information from the patient’s history, clinical examination, and PA radiography does not provide an accurate diagnosis. In such cases, the benefits for the individual patient outweigh the potential radiation exposure risks [50,62]. For most endodontic applications, limited FOV CBCT is preferred [63].

### 4.5. Clinical Implications

Clinicians may encounter situations where CBCT imaging is available without PA imaging, primarily when a medium or full FOV CBCT scan was performed for non-endodontic reasons, such as implant treatment planning, or when a patient is referred for endodontic treatment as the sole radiographic examination. The presence of intra-canal materials such as gutta-percha or metallic posts may produce streaks and dark bands that are artifacts on CBCT images, potentially reducing diagnostic efficacy [19,20,21,22].

**Figure 3:**
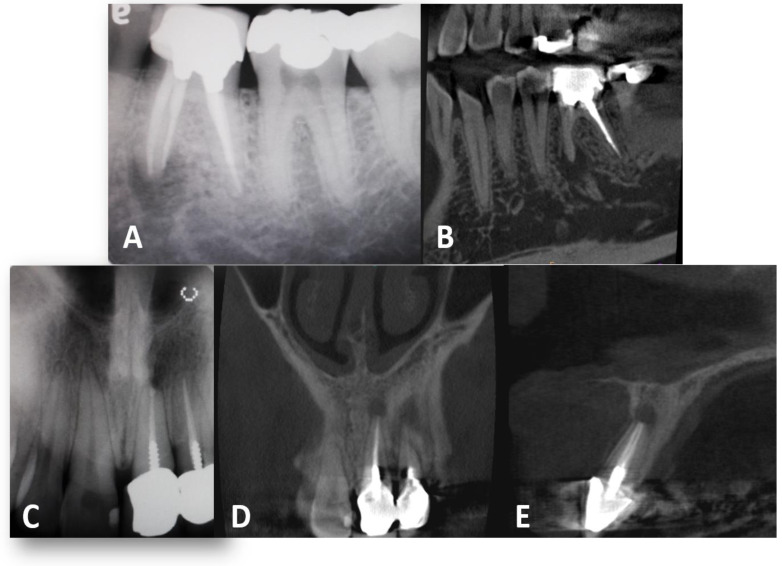
(**A**) A PA radiography of the first mandibular molar: post was not identified, there are amalgam dowels. (**B**) Frontal section from CBCT imaging of the same tooth. The observers identified a prefabricated post. (**C**) A PA radiography of the central incisor: a prefabricated was identified. (**D**,**E**) frontal and sagittal sections from CBCT imaging of the same tooth. The observers identified a cast post.

**Figure 4:**
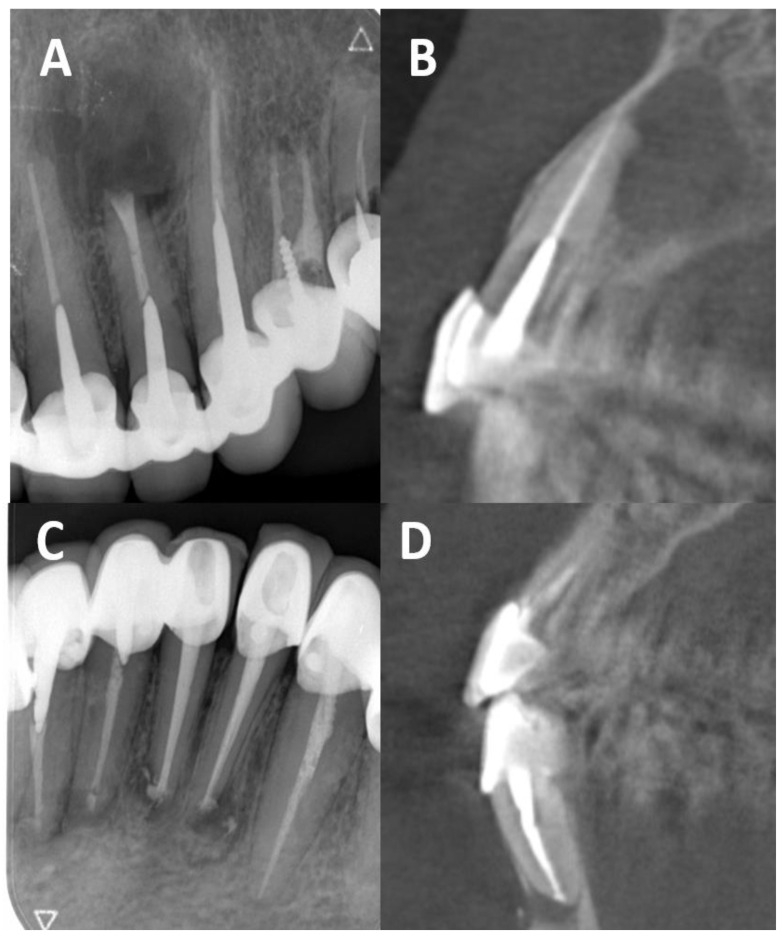
(**A**) A PA radiography of the upper canine: a cast post was identified. (**B**) Sagittal section from CBCT imaging of the same tooth. The observers identified a prefabricated post, because of a false sensation of a serrated outline of the post. (**C**) A PA radiography of the lower canine: a cast post was identified. (**D**) Sagittal section from CBCT imaging of the same tooth. The observers did not identify a post.

To our knowledge, no research has investigated the ability of CBCT to identify the presence or type of dental posts. Examination of the CBCT images in this study led to false-negative post identification, where 10.5% of teeth with a detected post on the PA images were invisible on CBCT. Examination of the CBCT images also led to false-positive post identification, where 17.6% of teeth without a post on the PA images were identified with a post on the CBCT images. The results were statistically significant and demonstrated that CBCT is not a proper imaging tool for dental post detection and post type identification. Figure 3 and Figure 4 provide examples of false identification.

The absence of PA images may lead to erroneous clinical decisions, such as opting for retreatment via FPDs when a prefabricated post is mistakenly identified on a CBCT scan, despite a cast post being present clinically. Conversely, FPDs may be unnecessarily removed if a post is falsely detected on CBCT imaging but not present clinically. In cases where a cast post is falsely identified, clinicians may lean towards surgical retreatment over the non-surgical retreatment option due to the perceived difficulty of its removal and associated risks. These scenarios can compromise the prognosis of the tooth, resulting in unnecessary treatments and financial and medico-legal consequences.

Existing guidelines specify the appropriate timing for conducting a CBCT scan but do not address situations where CBCT scans have already been performed. Additionally, not all clinicians strictly adhere to existing guidelines, as evidenced by practices in other dental fields [64,65,66]. The current study emphasizes the critical importance of conducting PA imaging even in cases where a CBCT scan has already been performed. Adjacent endodontically treated or multi-rooted teeth, comprising 13.8% of the teeth in the current study, may increase artifact disturbance on CBCT scans and reduce the CBCT’s ability to detect the presence of posts. The scatter effect complicates the accurate assessment of tooth conditions, potentially compromising the quality of decision-making. Therefore, future studies should investigate isolated single-root canal teeth versus multiple-rooted and adjacent teeth to gain a better understanding of the challenges of CBCT interpretation in real-life clinical scenarios affected by these scatter effects. Furthermore, future studies should include larger research samples, assess CBCT’s ability to detect fiber and titanium posts and consider various metal artifact reduction algorithms and image data processing methods. These efforts can enhance the clarity of CBCT’s diagnostic capability in accurately identifying dental posts.

In recent years, we have observed numerous clinical cases on social media where diagnoses and decisions were made solely based on CBCT scans. We cannot remain confined to academic theory; our goal is to prevent mistakes in real-life clinical situations. The authors have personally received many inquiries accompanied only by CBCT scans, requesting suitable treatment options. This research aims to address these widespread errors in clinical practices globally.

## 5. Conclusions

A CBCT image is an insufficient tool for the identification of stainless-steel prefabricated and cast posts under the limitations of this study.

PA image is highly recommended to achieve precise information about the post-endodontic restoration of teeth candidates for endodontic retreatment, even in cases wherein preoperative CBCT images exist.

## Figures and Tables

**Table 1:** The number of patients and their corresponding number of teeth included in this retrospective study.

5 teeth per patients	2
4 teeth per patients	2
3 teeth per patients	8
2 teeth per patients	17
1 tooth per patient	24
Total No. of patients	53

**Table 2:** Detection of a post on a PA radiograph.

A Post Was not Detected	A Prefabricated Post Was Detected	A Cast Post and Core Was Detected	Total No. of Teeth
34	43	24	101

**Table 3:** Detection of a post on a CBCT scan.

A Post Was not Detected	A Prefabricated Post Was Detected	A Cast Post and Core Was Detected	Post Was Detected. Its Type Could not Be Determined	Total No. of Teeth
36	46	14	5	101

## Data Availability

Dataset available on request from the authors.
